# Supplementary material for: Atrial TRPM2 Channel-Mediated Ca2+ Influx Regulates ANP Secretion and Protects Against Isoproterenol-Induced Cardiac Hypertrophy and Fibrosis
Source: Cells. 2025 Dec 22;15(1):24. doi: 10.3390/cells15010024 (PMC12785072; doi:10.3390/cells15010024)
Supplement: Supplementary file 1 [file cells-15-00024-s001.zip › cells-4015954-supplementary.pdf]

cells

Article

Atrial TRPM2 Channel-Mediated Ca^{2+} Influx Regulates ANP Secretion and Protects Against Isoproterenol-Induced Cardiac Hypertrophy and Fibrosis

Tomohiro Numata [1,*], Hideaki Tagashira [1], Kaori Sato-Numata [1], Meredith C Hermosura [2], Fumiha Abe [1], Ayako Sakai [1], Shinichiro Yamamoto [3] and Hiroyuki Watanabe [4]

1 Department of Integrative Physiology, Graduate School of Medicine, Akita University, 010-8543 Akita, Japan;
htagashira@med.akita-u.ac.jp (H.T.); satokao@med.akita-u.ac.jp (K.S.-N.); f_abe@hos.akita-u.ac.jp (F.A.); a.sakai@med.akita-u.ac.jp (A.S.)
2 John A. Burns School of Medicine, Honolulu, HI 96813, USA; hermosur@hawaii.edu
3 Faculty of Pharmaceutical Sciences, Teikyo Heisei University, 164-8530 Tokyo, Japan; s.yamamoto@thu.ac.jp
4 Department of Cardiovascular Medicine, Akita University Graduate School of Medicine, 010-8543 Akita, Japan;
hirow@doc.med.akita-u.ac.jp
* Correspondence: numata@med.akita-u.ac.jp; Tel.: +81-18-884-6272

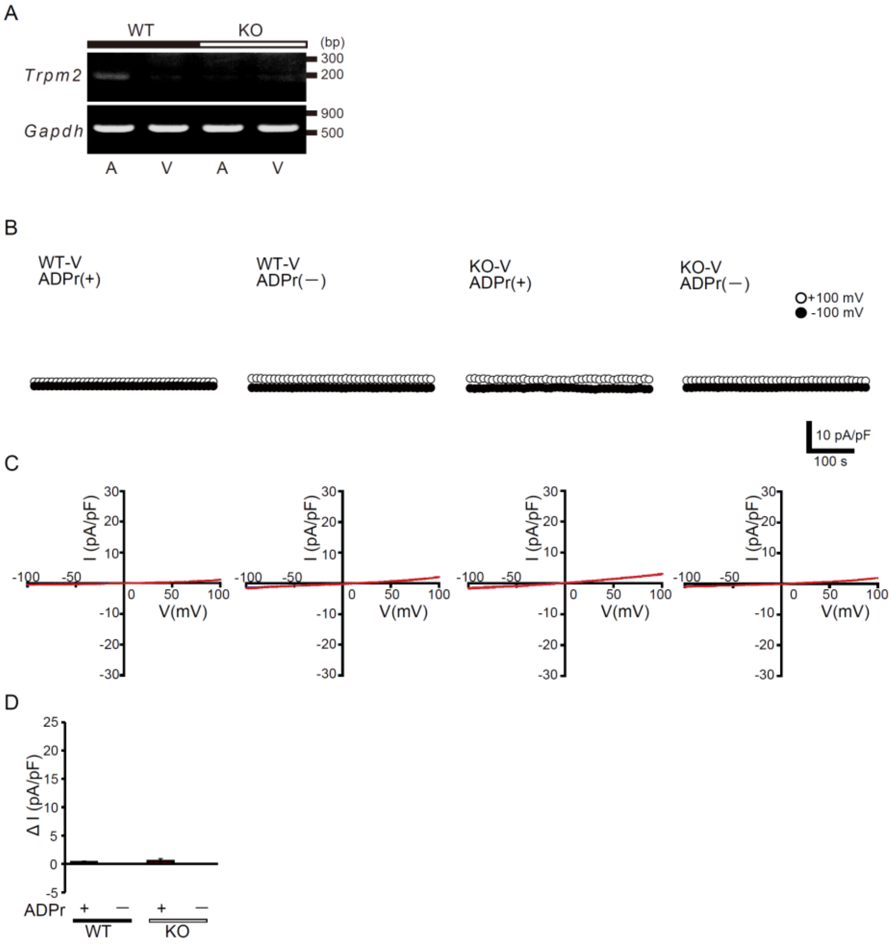

 https://doi.org/10.3390/cells15010024

Figure S1. Ventricular TRPM2 expression and lack of ADPr-evoked currents. A) Conventional RT-PCR for *Trpm2* and *Gapdh* using RNA from atria (A) and ventricles (V) of wild-type (WT) and TRPM2 knockout (KO) mouse hearts. Representative gel image is shown (n = 3 independent hearts per genotype); right margin indicates DNA ladder sizes (bp). Gapdh served as the internal control. A, atria; V, ventricle; WT, wild-type; KO, TRPM2$^{-/-}$. (B) Whole-cell patch-clamp recordings from isolated ventricular myocytes. Time courses of currents at +100 mV (open circles) and −100 mV (filled circles) are shown for WT and KO cells dialyzed with intracellular ADP-ribose (ADPr, 0.5 mM; ADPr (+)) or without ADPr (ADPr (−)). The dotted horizontal line indicates the zero-current baseline. (C) Corresponding I–V relationships (ramps from +100 to −100 mV) at steady state for the four conditions (WT-V ADPr(+), WT-V ADPr(−), KO-V ADPr(+), KO-V ADPr(−)). (D) Summary of ADPr-activated current density (ΔI at +100 mV; pA/pF) in ventricular myocytes. Data are presented as mean ± SEM (n = 6-7). Group differences were analyzed using Student's t-test. No significant differences were observed among groups, confirming the absence of functional TRPM2 currents in ventricular myocytes.

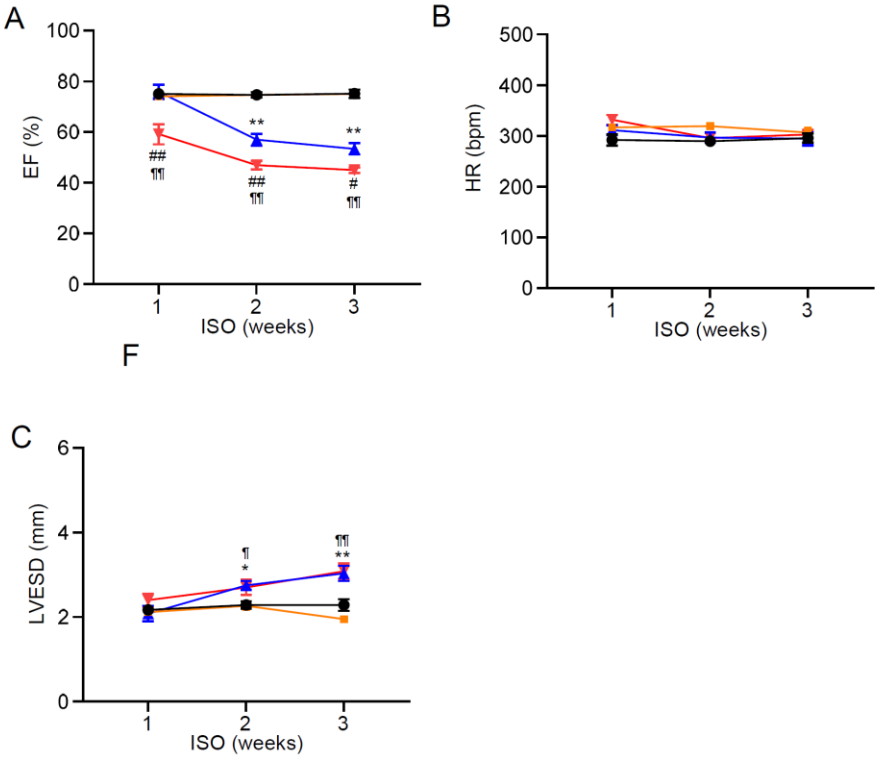

Figure S2. Additional echocardiographic indices corresponding to Figure 2. Time-course data (weeks 1–3 of ISO) in WT, TRPM2$^{-/-}$ (KO), WT+ISO, and TRPM2$^{-/-}$+ISO mice. (A) Ejection fraction (EF, %) over time. (B) Heart rate (HR, bpm) over time. (C) Left-ventricular end-systolic diameter (LVESD, mm) over time. All values are presented as mean ± SEM (n = 5–6). Group differences were analyzed using ANOVA. $^{*}p < 0.05$, $^{**}p < 0.01$ WT vs WT+ISO; $^{¶}p < 0.05$, $^{¶¶}p < 0.01$ KO vs KO+ISO; $^{\#}p < 0.05$, $^{\#\#}p < 0.01$ WT + ISO + Veh vs KO + ISO.

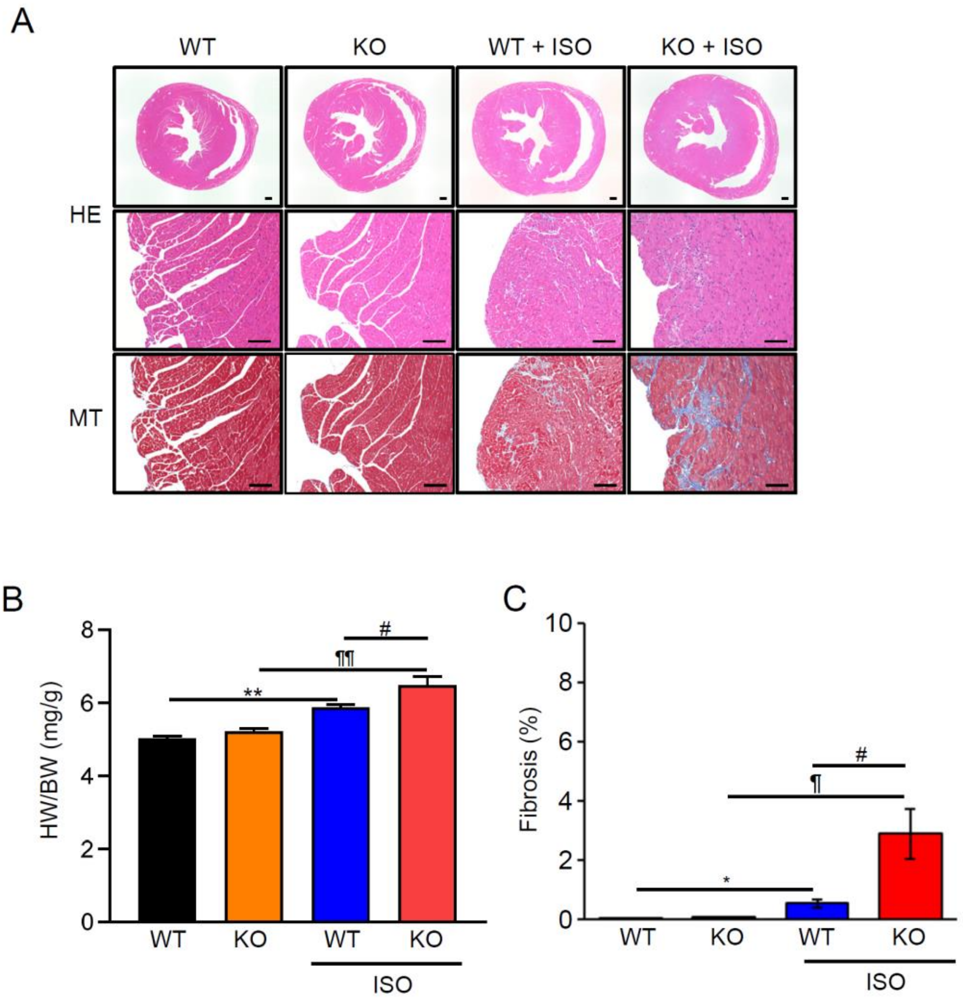

Figure S3. Additional histology and quantification corresponding to Figure 3A) Representative hematoxylin–eosin (HE; whole heart and higher magnification) and Masson's trichrome (MT) sections. (B) Heart weight–to–body weight (HW/BW, mg·g^{-1}) for each group (WT, TRPM2$^{-/-}$ (KO), WT + ISO, KO + ISO) (n = 8-9). (C) Fibrosis quantification (% MT-positive area) (n = 6). Scale bars: 100 μm (whole-heart HE inset), 500 μm (HE/MT panels). Data are presented as mean ± SEM. Statistics: One-way ANOVA followed by appropriate post hoc testing. $^{*}p < 0.05$, $^{**}p < 0.01$ WT vs WT + ISO ± ANP; $^{¶}p < 0.05$, $^{¶¶}p < 0.01$ KO vs KO + ISO; $^{\#}p < 0.05$ WT + ISO vs KO + ISO.

Table S1. Primer sets used for RT-PCR/qPCR. Gene symbols refer to mouse transcripts (italicized in text). Primer sequences are listed 5′→3′; amplicon size indicates the expected product length in base pairs (bp). *Nppa* encodes atrial natriuretic peptide (ANP) and *Npr1* encodes the ANP receptor NPR-A.

Gene (mouse)	Forward primer (5'→3')	Reverse primer (5'→3')	Amplicon (bp)
Trpm2	CATCGTGAAGCGGATGATGAAG	TGTAGGTTGGGATCTGGCCAAA	182
Gapdh	AACTTTGGCATTGTGGAAGG	TGGGGGCCGAGTTGGGATAG	578
Nppa (ANP)	GGGGGTAGGATTGACAGGAT	ACACACCACAAGGGCTTAGG	139
Npr1 (NPR-A)	CGAAGCTTCCAAGGTGTGACAGG	GACACAGCCATCAGCTCCTGGG	152
